# CD40 ligand induces RIP1-dependent, necroptosis-like cell death in low-grade serous but not serous borderline ovarian tumor cells

**DOI:** 10.1038/cddis.2015.229

**Published:** 2015-08-27

**Authors:** X Qiu, C Klausen, J-C Cheng, P C K Leung

**Affiliations:** 1 Department of Obstetrics and Gynaecology, Child & Family Research Institute, University of British Columbia, Vancouver, British Columbia V5Z 4H4, Canada

## Abstract

Ovarian high-grade serous carcinomas (HGSCs) and invasive low-grade serous carcinomas (LGSCs) are considered to be distinct entities. In particular, LGSCs are thought to arise from non-invasive serous borderline ovarian tumors (SBOTs) and show poor responsiveness to conventional chemotherapy. The pro-apoptotic effects of CD40 ligand (CD40L) have been demonstrated in HGSC, though the underlying mechanisms are not fully understood. Conversely, the therapeutic potential of the CD40L-CD40 system has yet to be evaluated in LGSC. We now show that CD40 protein is focally expressed on tumor cells in two of five primary LGSCs compared with no expression in eight primary SBOTs. Treatment with CD40L or agonistic CD40 antibody decreased the viability of LGSC-derived MPSC1 and VOA1312 cells, but not SBOT3.1 cells. Small interfering RNA (siRNA) targeting CD40 was used to show that it is required for these reductions in cell viability. CD40L treatment increased cleaved caspase-3 levels in MPSC1 cells though, surprisingly, neither pan-caspase inhibitor nor caspase-3 siRNA reversed or even attenuated CD40L-induced cell death. In addition, CD40-induced cell death was not affected by knockdown of the mitochondrial proteins apoptosis-inducing factor (AIF) and endonuclease G (EndoG). Interestingly, CD40L-induced cell death was blocked by necrostatin-1, an inhibitor of receptor-interacting protein 1 (RIP1), and attenuated by inhibitors of RIP3 (GSK'872) or MLKL (mixed lineage kinase domain-like; necrosulfonamide). Our results indicate that the upregulation of CD40 may be relatively common in LGSC and that CD40 activation induces RIP1-dependent, necroptosis-like cell death in LGSC cells.

Epithelial ovarian cancer accounts for approximately 90% of all ovarian malignancies and is the leading cause of gynecological cancer death in developed countries.^1, 2^ Recently, differences in molecular alterations and clinicopathological features have established a dualistic model dividing ovarian serous carcinomas into high-grade serous carcinoma (HGSC) and low-grade serous carcinoma (LGSC) subtypes. HGSCs are more common and are thought to develop directly from the ovarian surface epithelium or from serous tubal intra-epithelial carcinomas in the fallopian tube. In contrast, LGSCs are rare and are generally considered to develop from benign serous cystadenomas through serous borderline ovarian tumors (SBOT). SBOTs are slow-growing, non-invasive epithelial neoplasms that have a better prognosis compared with other types of ovarian cancer.^3, 4, 5^ Our previous studies have shown that the inhibition of p53 or treatment of epidermal growth factor or transforming growth factor-*β*1 increases SBOT cell invasion by inducing epithelial–mesenchymal transition, which suggests a possible mechanism that mediates the progression from SBOT to LGSC.^6, 7, 8, 9^ However, many of SBOTs recur as LGSCs that display poor responsiveness to conventional chemotherapy and for which survival rates are <50%.^1, 3, 10^ Thus, the development of novel, targeted therapeutic strategies is likely required to significantly improve patient survival.

CD40, a transmembrane glycoprotein belonging to the tumor necrosis factor receptor superfamily, is expressed by a wide range of cell types including immune, endothelial and epithelial cells. Engagement of CD40 with its ligand, CD40L, has been shown to have important roles in a variety of physiological and pathological processes, especially in immunity.^11, 12^ In addition, CD40 expression has been demonstrated in several types of cancer, including colon, lung, cervical, bladder and prostate cancer.^13^ However, reported functions of CD40 in tumor cells vary, with both pro-apoptotic and anti-proliferative effects observed depending on the cellular context.^14, 15, 16^ Alternatively, some studies have shown that CD40 activation may promote the neoplastic transformation and growth of normal cells.^17, 18, 19^ Expression of CD40 has been demonstrated in ovarian cancer cell lines and tumor samples, but not in normal ovarian tissue, suggesting that CD40 may have an important role in ovarian tumors.^20, 21, 22, 23, 24^ Indeed, CD40L-CD40 signaling has been shown to induce growth-inhibitory effects in HGSC cells,^20, 21, 23, 24, 25^ however, the therapeutic potential of CD40 in LGSC and SBOT has not been evaluated.

In the present study, we report for the first time elevated CD40 expression in a significant proportion of LGSCs compared with SBOTs. Moreover, CD40 expression is elevated in LGSC-derived MPSC1 and VOA1312 cells compared with SBOT3.1 cells, and CD40 activation induces cell death via CD40 only in LGSC-derived cells. Neither pan-caspase inhibitor nor caspase-3 small interfering RNA (siRNA) has any effect on CD40L-induced MPSC1 cell death. Moreover, CD40L-induced cell death was unaffected by individual or combined knockdown of the mitochondrial proteins apoptosis-inducing factor (AIF) and endonuclease G (EndoG). Interestingly, our results suggest that receptor-interacting protein 1 (RIP1), RIP3 and MLKL are involved in CD40-induced MPSC1 cell death. These results demonstrate that CD40 induces RIP1-dependent, necroptosis-like cell death in LGSC cells.

## Results

### Expression of CD40 in SBOT- and LGSC-derived cell lines and primary tumor samples

**Figure 1:**
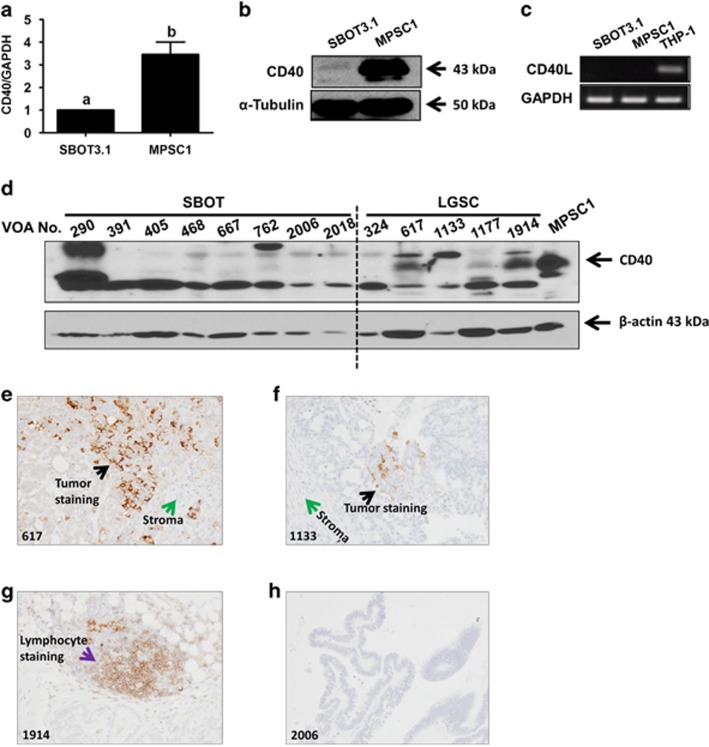
Expression of CD40 in SBOT- and LGSC-derived cell lines and primary tumor samples. (**a** and **b**) RT-qPCR and western blot were used to measure endogenous CD40 mRNA and protein levels in SBOT-derived SBOT3.1 cells and LGSC-derived MPSC1 cells. Quantitative results are expressed as the mean±S.E.M. of at least three independent passages and values without a common letter are significantly different (*P*<0.05). (**c**) Endogenous CD40L mRNA levels in SBOT3.1 and MPSC1 cells were measured by RT-qPCR. THP-1 human acute monocytic leukemia cells were used as a positive control and RT-qPCR products were analyzed by agarose gel electrophoresis. (**d**) Western blot was used to measure endogenous CD40 protein levels in MPSC1 cells (positive control) and frozen tissues from primary SBOTs and LGSCs (labeled with 'VOA#'). (**e**–**h**) Representative CD40 immunostaining results for matched sections from the patient samples analyzed by western blot

A previous study analyzing the DNA methylation profiles of ovarian serous neoplasms indicated that *CD40* is hypomethylated in LGSCs compared with SBOTs, suggesting the expression of CD40 may be higher in LGSCs than in SBOTs.^26^ To test this hypothesis, we examined CD40 expression levels in SBOT-derived SBOT3.1 cells and LGSC-derived MPSC1 cells. CD40 mRNA (Figure 1a) and protein (Figure 1b) levels were higher in MPSC1 cells than in SBOT3.1 cells. As many CD40-expressing cells also express CD40L, we also examined the expression of CD40L in these two cell lines. As shown in Figure 1c, CD40L mRNA was undetectable in both SBOT3.1 and MPSC1 cells. These results suggest that both SBOT3.1 and MPSC1 cells express CD40, but that CD40 levels are much higher in LGSC-derived MPSC1 cells.

Next, we used western blot to measure CD40 protein levels in frozen tissues from eight SBOTs and five LGSCs. As shown in Figure 1d, CD40 protein levels were elevated in three of five LGSC samples compared with weak or no expression in the SBOT samples. To confirm CD40 expression in LGSC tumor cells, we immunostained matching sections from all eight SBOTs and five LGSCs. Focal, positive staining for CD40 was observed in tumor cells from two of five LGSC samples (Figures 1e and f). Interestingly, one of the LGSC samples with CD40-negative tumor cells contained multiple CD40-positive lymphoid follicles (Figure 1g), which are likely the cause of its positivity in western blot. Unlike the LGSC samples, all SBOT samples were negative for CD40 (Figure 1h).

### CD40 activation induces cell death in LGSC-derived cells but not SBOT3.1 cells

**Figure 2:**
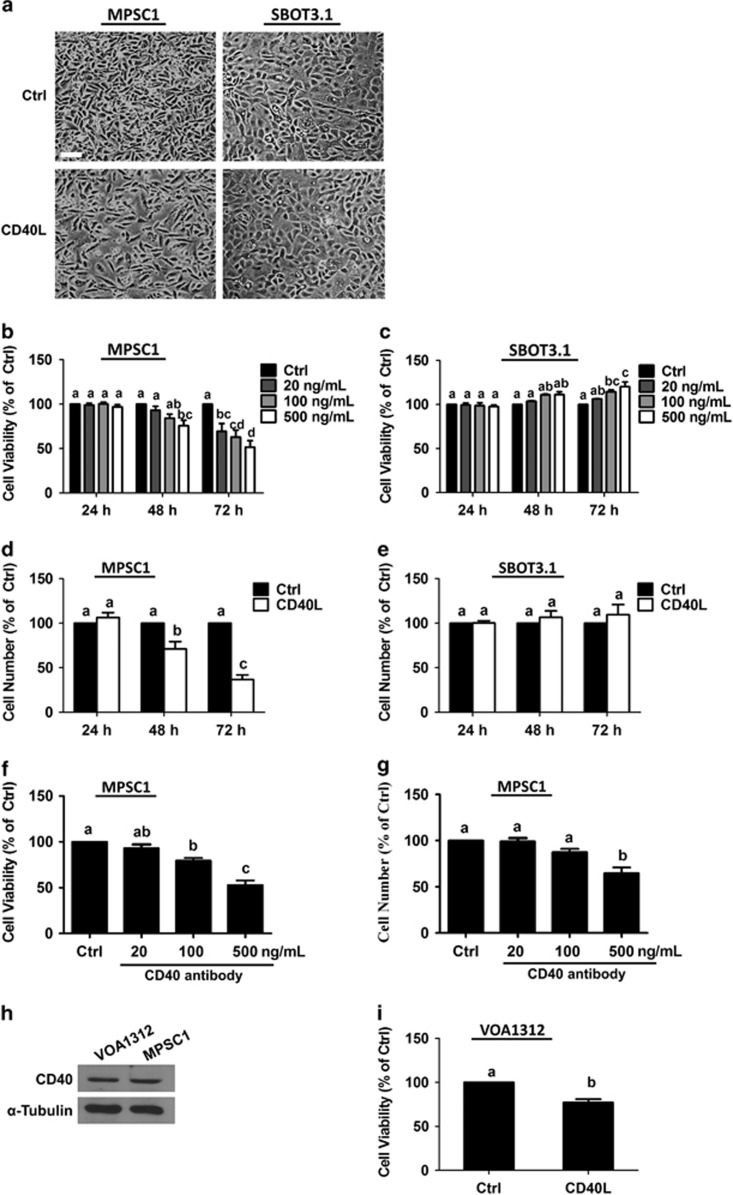
CD40 activation induces cell death in MPSC1 not SBOT3.1 cells. (**a**) Cells were treated for 48 h with vehicle control (Ctrl) or 500 ng/ml recombinant human CD40L, and cell morphology was assessed by phase contrast microscopy. Scale bar: 200 *μ*M. (**b** and **c**) Cells were treated for 24, 48 or 72 h with vehicle control (Ctrl) or different concentrations of CD40L and cell viability was examined by the MTT assay. (**d** and **e**) Alternatively, viable cell numbers were measured by Trypan blue exclusion assay following treatment for 24, 48 or 72 h with vehicle control or 500 ng/ml CD40L. (**f** and **g**) Cells were treated for 72 h with vehicle control or different concentrations of agonistic CD40 antibody and cell viability (**f**) and cell number (**g**) were analyzed by MTT and Trypan blue exclusion assays, respectively. (**h**) Western blot was used to measure endogenous CD40 protein levels in VOA1312 and MPSC1 cells. (**i**) VOA1312 cells were treated for 72 h with vehicle control or 500 ng/ml CD40L and cell viability was examined by the MTT assay. Results are expressed as the mean±S.E.M. of at least three independent experiments. Values without a common letter are significantly different (*P*<0.05)

Growth-inhibitory and pro-apoptotic effects of CD40 activation have previously been demonstrated in HGSC cells,^20, 21, 23, 24, 25^, however, its effects on SBOT and LGSC cells are unknown. To investigate the effects of CD40L on SBOT and LGSC, SBOT3.1 and MPSC1 cells were treated for 48 h with 500 ng/ml recombinant human CD40L and morphology was assessed by phase contrast microscopy. As shown in Figure 2a, treatment with CD40L did not affect the morphology of SBOT3.1 cells; however, it significantly decreased the number of MPSC1 cells, suggesting potential pro-apoptotic effects of CD40L in MPSC1 cells. To expand on these findings, MPSC1 and SBOT3.1 cells were treated for 24, 48 or 72 h with different concentrations of CD40L (20, 100 or 500 ng/ml) and cell viability was examined by the MTT assay (Figures 2b and c). CD40L treatment did not diminish SBOT3.1 cell viability, but it reduced that of MPSC1 cells in both a time- and concentration- dependent manner, with the most significant reductions occurring 72 h after treatment. To further confirm these effects on cell viability, we measured viable cell numbers by Trypan blue exclusion assay following treatment with 500 ng/ml CD40L for 24, 48 or 72 h. In agreement with our MTT results, CD40L treatment induced time-dependent reductions in viable MPSC1 cell numbers but did not alter SBOT3.1 cell viability (Figures 2d and e). Moreover for both methods, the number of viable cells at 72 h was significantly lower than that at 24 or 48 h, indicating that CD40L-induced decreases in MPSC1 cell viability are mediated, at least in part, by increased cell death. Interestingly, we do not believe that low CD40 levels are entirely responsible for the lack of response in SBOT3.1 cells, because these cells can produce CD40L-induced increases in ERK1/2 phosphorylation that are comparable with those observed in MPSC1 cells (Supplementary Figure 1).

**Figure 3:**
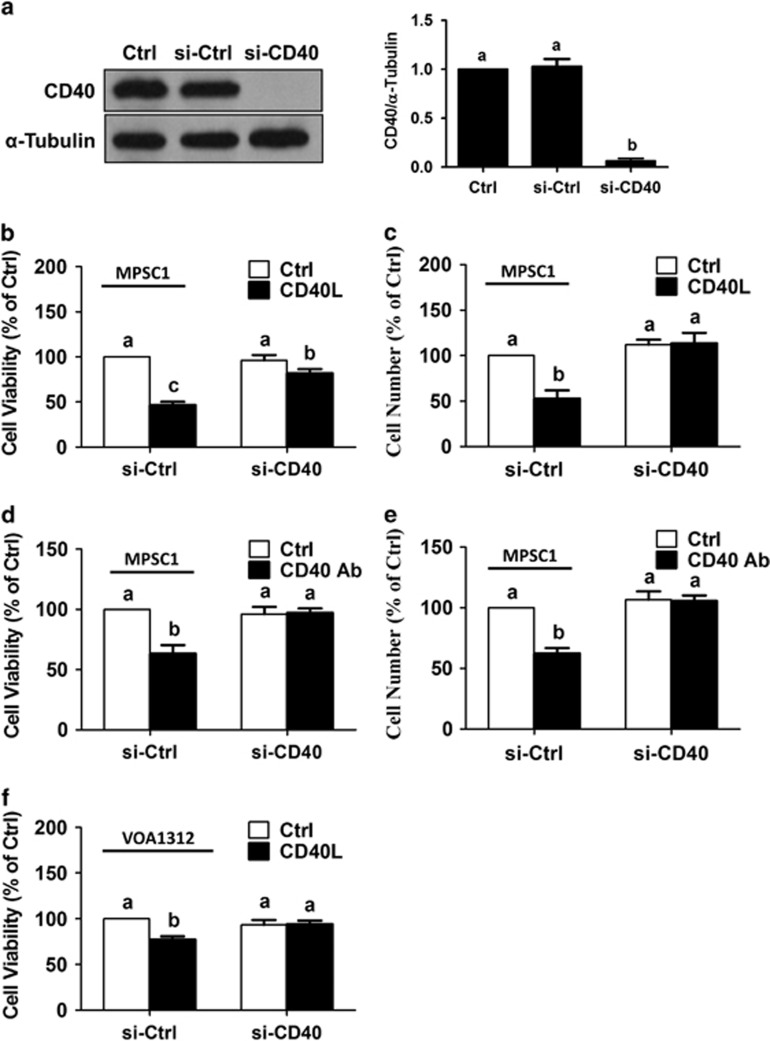
CD40 is required for CD40L-induced cell death. (**a**) MPSC1 cells were transfected for 24 h with 50 nM control siRNA (si-Ctrl) or CD40 siRNA (si-CD40) and knockdown efficiency was examined by western blot. Following transfection as described in (**a**), MPSC1 cells were treated for 72 h with vehicle control (Ctrl) or 500 ng/ml CD40L (**b** and **c**) or agonistic CD40 antibody (**d** and **e**) and cell viability (**b** and **d**) and cell number (**c** and **e**) were analyzed by MTT and Trypan blue exclusion assays, respectively. (**f**) VOA1312 cells were transfected for 24 h with 50 nM control siRNA or CD40 siRNA, treated for another 72 h with vehicle control or CD40L (500 ng/ml), and cell viability was examined by the MTT assay. Results are expressed as the mean±S.E.M. of at least three independent experiments. Values without a common letter are significantly different (*P*<0.05)

Next, we examined the effects of CD40 ligation on MPSC1 cell viability by treating the cells with agonistic CD40 antibody. As shown in Figures 2f and g, similar reductions in MPSC1 cell viability or viable cell numbers were observed following treatment for 72 h with 500 ng/ml agonistic CD40 antibody. To further confirm that CD40 activation reduces the viability of LGSC cells, we examined the effects of CD40L on LGSC-derived VOA1312 cells^27^ which have CD40 protein levels similar to those of MPSC1 cells (Figure 2h). As shown in Figure 2i, VOA1312 cell viability was reduced following treatment for 72 h with 500 ng/ml CD40L. To determine whether CD40 is required for CD40L-induced cell death in LGSC cells, we examined the effects of CD40L on cell viability following siRNA-mediated knockdown of endogenous CD40. Pre-treatment of MPSC1 cells for 24 h with CD40 siRNA significantly reduced CD40 protein levels (Figure 3a), and reversed the effects of subsequent treatment for 72 h with 500 ng/ml of either CD40L (Figures 3b and c) or agonistic CD40 antibody (Figures 3d and e) on cell viability as assessed by MTT or Trypan blue exclusion assays. In addition, knockdown of CD40 also reversed the effects of CD40L on cell viability in VOA1312 cells (Figure 3f).

### Caspase-3 is activated during CD40L-induced MPSC1 cell death

**Figure 4:**
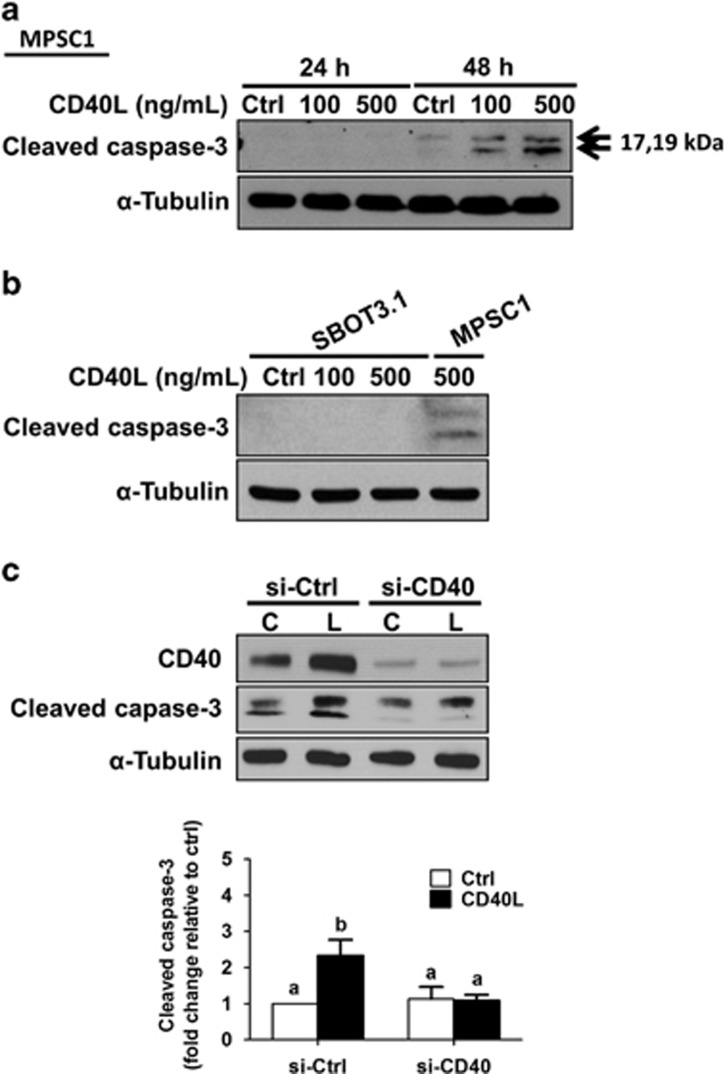
Caspase-3 is activated during CD40L-induced MPSC1 cell death. (**a**) Cleaved caspase-3 levels were measured by western blot following treatment of MPSC1 cells for 24 or 48 h with vehicle control (Ctrl) or CD40L (100 or 500 ng/ml). (**b**) SBOT3.1 cells were treated for 48 h with vehicle control (Ctrl) or CD40L and cleaved caspase-3 levels were measured by western blot. (**c**) MPSC1 cells were transfected for 24 h with 50 nM control siRNA (si-Ctrl) or CD40 siRNA (si-CD40) and then treated for another 48 h with vehicle control (Ctrl) or CD40L (500 ng/ml). CD40 and cleaved caspase-3 were analyzed by western blot and quantified cleaved caspase-3 levels (right) are expressed as the mean±S.E.M. of at least three independent experiments. Values without a common letter are significantly different (*P*<0.05)

Next, we sought to determine whether apoptosis, a well-known form of programmed cell death, was involved in CD40L-induced MPSC1 cell death. Cleavage and activation of caspase-3, a critical executioner caspase, is often associated with apoptotic cell death.^28, 29^ Thus, we used western blot to measure cleaved caspase-3 levels in MPSC1 cells following treatment for 24 or 48 h with CD40L (100 or 500 ng/ml). CD40L treatment increased the levels of cleaved caspase-3 after 48 h in MPSC1 cells (Figure 4a). Consistent with our cell viability results, treatment of SBOT3.1 cells for 48 h with CD40L (100 or 500 ng/ml) did not alter the levels of cleaved caspase-3 (Figure 4b). Importantly, CD40L-induced increases in cleaved caspase-3 levels were abolished by pre-treatment of MPSC1 cells for 24 h with CD40 siRNA (Figure 4c). These results indicate that CD40L/CD40 signaling can activate caspase-3 in LGSC-derived MPSC1 cells but not SBOT3.1 cells.

### CD40L-induced MPSC1 cell death is caspase-independent

**Figure 5:**
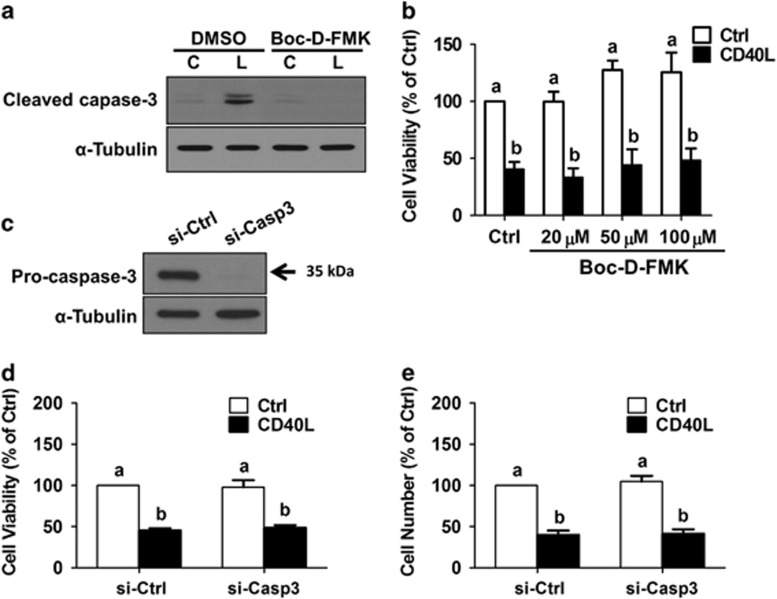
CD40L-induced MPSC1 cell death is caspase-independent. (**a**) Cells were pre-treated for 2 h with or without 20 *μ*M Boc-D-FMK and then treated for 48 h with vehicle control (Ctrl) or CD40L (500 ng/ml). Cleaved caspase-3 levels were measured by western blot. (**b**) Cell viability was measured by MTT assay following treatment for 72 h with vehicle control or CD40L (500 ng/ml) in the presence or absence of different concentrations of Boc-D-FMK (20, 50 or 100 *μ*M). (**c**) Cells were transfected for 24 h with 50 nM control siRNA (si-Ctrl) or caspase-3 siRNA (si-Casp3) and knockdown efficiency was examined by western blot. Following transfection as described in (**c**), cells were treated for 72 h with vehicle control or CD40L (500 ng/ml) and cell viability (**d**) and cell number (**e**) were analyzed by MTT and Trypan blue exclusion assays, respectively. Results are expressed as the mean±S.E.M. of at least three independent experiments. Values without a common letter are significantly different (*P*<0.05)

To determine whether activated caspase-3 is directly involved in CD40L-induced cell death, MPSC1 cell viability and cleaved caspase-3 levels were examined in the presence or absence of an irreversible pan-caspase inhibitor (Boc-D-FMK). Pre-treatment for 2 h with 20 *μ*M Boc-D-FMK completely blocked CD40L-induced increases in cleaved caspase-3 levels (Figure 5a). Surprisingly, pre-treatment with Boc-D-FMK (20, 50 or 100 *μ*M) did not reverse, or even attenuate, the effects of CD40L (500 ng/ml, 72 h) on cell viability as measured by MTT assay (Figure 5b). To confirm these findings, we examined the effects of CD40L on MPSC1 cell viability following siRNA-mediated knockdown of caspase-3. Pre-treatment for 24 h with caspase-3 siRNA significantly reduced pro-caspase-3 protein levels (Figure 5c), but did not alter the effects of subsequent treatment with CD40L (500 ng/ml, 72 h) on cell viability as measured by MTT or Trypan blue exclusion assays (Figures 5d and e). These results suggest that CD40L-induced cell death in LGSC-derived MPSC1 cells is caspase-independent.

### CD40L induces RIP1-dependent, necroptosis-like cell death in MPSC1 cells

**Figure 6:**
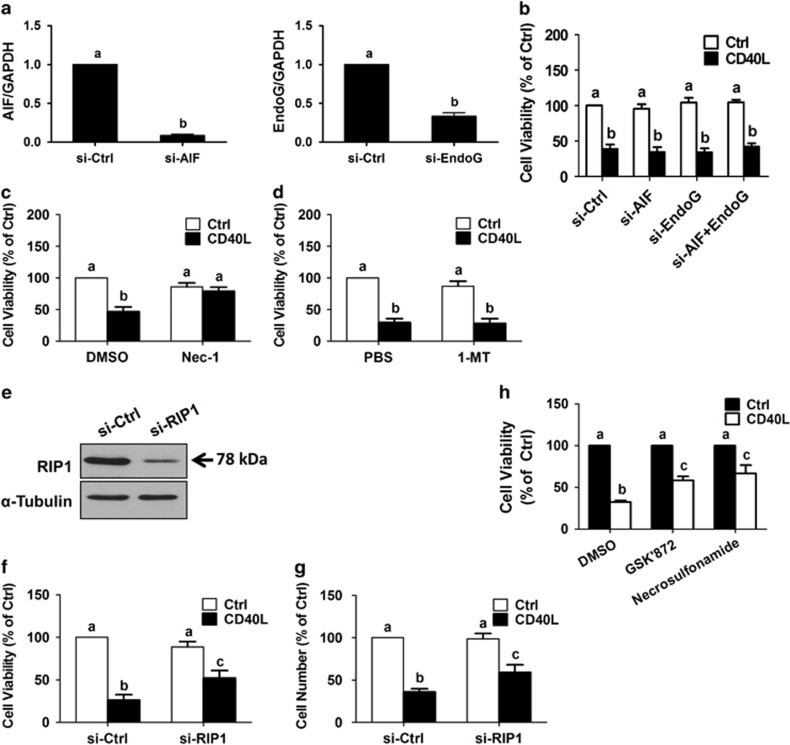
CD40L induces mitochondria-independent but RIP1-dependent cell death in MPSC1 cells. (**a**) Cells were transfected for 24 h with 50 nM control siRNA (si-Ctrl), AIF siRNA (si-AIF) or EndoG siRNA (si-EndoG), and knockdown efficiency was examined by RT-qPCR. (**b**) Cells were transfected for 24 h with the indicated siRNAs alone or in combination prior to being treated for 72 h with vehicle control (Ctrl) or CD40L (500 ng/ml). Cell viability was measured by MTT assay. (**c** and **d**) Cell viability was measured by MTT assay following treatment for 72 h with vehicle control or CD40L (500 ng/ml) in the presence or absence of 150 nM necrostatin-1 (**c**) or 1-MT (D). (**e**) Cells were transfected for 24 h with 50 nM control siRNA or RIP1 siRNA (si-RIP1), and knockdown efficiency was examined by western blot. Following transfection as described in (**e**), transfected cells were treated for 72 h with vehicle control or CD40L (500 ng/ml), and cell viability (**f**) and cell number (**g**) were analyzed by MTT and Trypan blue exclusion assays, respectively. (**h**) Cell viability was measured by MTT assay following treatment for 72 h with vehicle control or CD40L (500 ng/ml) in the presence or absence of 6 *μ*M GSK'872 or 3 *μ*M necrosulfonamide. Results are expressed as the mean±S.E.M. from at least three independent experiments. Values without a common letter are significantly different (*P*<0.05)

Mitochondria are central to the control of cell death, and mitochondria-dependent cell death is characterized by the release of mitochondrial proteins into the cytoplasm that are capable of inducing caspase-dependent or caspase-independent cell death.^30, 31^ AIF and EndoG are mitochondrial proteins that are known to translocate to the nucleus and cause chromatin condensation and DNA cleavage in a caspase-independent manner.^32, 33^ To determine whether AIF and/or EndoG are required for CD40L-induced MPSC1 cell death, we examined the effects of CD40L on cell viability following siRNA-mediated knockdown of endogenous AIF and/or EndoG. Pre-treatment for 24 h with AIF and/or EndoG siRNA significantly reduced AIF and EndoG mRNA levels (Figure 6a), but did not alter the effects of subsequent treatment with CD40L (500 ng/ml, 72 h) on cell viability as measured by MTT assay (Figure 6b). These results suggest that CD40L-induced cell death in LGSC-derived MPSC1 cells is mitochondria-independent.

RIP1 and RIP3 kinases have emerged as important regulators of a form of caspase-independent cell death referred to as necroptosis.^34, 35^ To determine whether RIP1 is required for CD40L-induced cell death, MPSC1 cell viability was measured in the presence or absence of an allosteric inhibitor of RIP1 (necrostatin-1). Interestingly, pre-treatment for 2 h with 150 nM necrostatin-1 completely blocked CD40L-induced reductions in cell viability as measured by MTT assay (Figure 6c). However, several studies have shown that necrostatin-1 also inhibits indoleamine-2,3-dioxygenase.^36, 37^ To exclude the possible involvement of indoleamine-2,3-dioxygenase, MPSC1 cells were pre-treated for 2 h with the indoleamine-2,3-dioxygenase inhibitor 1-methyl-L-tryptophan (1-MT, 150 nM) prior to being treated for 72 h with 500 ng/ml CD40L. As shown in Figure 6d, CD40L-induced reductions in cell viability were not affected by treatment with 1-MT. To further confirm the involvement of RIP1 in CD40L-induced cell death, we examined the effects of CD40L on cell viability following siRNA-mediated knockdown of endogenous RIP1. Pre-treatment for 24 h with RIP1 siRNA significantly reduced RIP1 protein levels (Figure 6e), and partially reversed the effects of subsequent treatment with CD40L (500 ng/ml, 72 h) on cell viability as assessed by MTT or Trypan blue exclusion assays (Figures 6f and g). Increasing evidence suggests that interactions between RIP1 and RIP3 are crucial to necrosome formation, and that MLKL (mixed lineage kinase domain-like), a critical substrate of RIP3, is a key effector of necroptosis.^35, 38^ To investigate the involvement of RIP3 and MLKL in CD40-induced cell death, MPSC1 cell viability was measured in the presence or absence of specific inhibitors of RIP3 (GSK'872) or MLKL (necrosulfonamide). As shown in Figure 6h, pre-treatment for 2 h with GSK'872 or necrosulfonamide partially reversed CD40L-induced reductions in cell viability as measured by MTT assay. Collectively, these data suggest that CD40 induces RIP1-dependent, necroptosis-like cell death in MPSC1 cells.

## Discussion

Invasive LGSCs display poor responsiveness to conventional chemotherapy, thus novel therapeutic strategies are urgently required to improve patient survival. We now show that CD40 protein is expressed in a significant proportion of LGSCs, perhaps as many as half, compared with weak or no expression in SBOTs. These results are consistent with a previous study suggesting hypomethylation of *CD40* in LGSCs compared with SBOTs,^26^ though future studies will be required to confirm an epigenetic basis for elevated CD40 expression in LGSCs. Importantly, we show for the first time that treatment with CD40L or agonistic CD40 antibody induces cell death in LGSC-derived cells via CD40 activation. Thus, recombinant human CD40L or agonistic CD40 antibody could represent novel treatment options for patients with LGSC displaying elevated CD40. Anti-tumor effects for CD40L-CD40 signaling have been shown in various types of CD40-positive tumors, with direct apoptotic cell killing accounting for much of the response.^39, 40, 41, 42, 43^ Indeed, recombinant CD40L treatment of CD40-positive HGSC xenografts in severe combined immunodeficient mice induced significant apoptosis and tumor destruction, and increased the efficacy of suboptimal doses of cisplatin.^25^

In addition to directly inducing tumor cell death, CD40-targeted treatments can stimulate general immune activation and have demonstrated utility as cancer immunotherapies, for which CD40 expression on tumor cells is not necessary.^44^ Activation of CD40 on antigen-presenting cells licenses them to stimulate T-killer cells to exert killing responses.^45^ Several studies have demonstrated the effectiveness of CD40 ligation in triggering the elimination of tumor cells by T-killer cells.^46, 47^ Moreover, CD40-induced anti-tumor effects have also been shown to involve activated macrophages^48, 49^ as well as B cells and natural killer cells.^50, 51, 52^ Interestingly, our immunostaining results show that some primary LGSCs with CD40-negative tumor cells contain CD40-positive lymphoid cells. In this context, patients with SBOT or LGSC displaying weak or no expression of CD40 may still benefit from CD40-targeted therapies owing to the enhancement of antigen-presenting cell function and the activation of T cells and natural killer cells. Patients with CD40-positive LGSC could also benefit from enhanced immune activation, including opsonization effects if treated with anti-CD40 antibody. Future studies investigating the potential of CD40-targeted therapies on CD40-positive and -negative LGSCs *in vivo* will be of great interest.

Cell death can occur in several ways including necrosis, apoptosis and necroptosis. Apoptosis, a form of programmed cell death, is accompanied by a host of morphological and biochemical features, including plasma membrane blebbing, cell shrinkage, chromatin condensation, apoptotic bodies, DNA fragmentation and phosphatidylserine exposure.^53, 54^ Caspases are the primary effectors of apoptotic cell death and caspase-3 is considered an important executioner owing to its activation of the endonuclease CAD, which can degrade chromosomal DNA.^55^ Interestingly, though treatment with CD40L resulted in caspase-3 activation, it was not required for CD40L-induced MPSC1 cell death. Moreover, redundant effects from other caspases are unlikely because CD40L-induced cell death was unaffected by pre-treatment with the broad-spectrum caspase inhibitor Boc-D-FMK. Interestingly, beyond their critical roles in apoptosis, increasing evidence suggests a variety of non-apoptotic functions of caspases.^56, 57^ For example, caspase-3 is transiently activated and functions as a key protease in the processes of erythroid differentiation^58^ and maturation.^59^ Caspase-3 has also been shown to inhibit B-cell cycling,^60^ promote adult hematopoietic stem cell quiescence^61^ and mediate embryonic stem cell differentiation.^62^ Thus, CD40L-induced caspase-3 activation in LGSC cells could indicate additional non-apoptotic roles that warrant further investigation.

Caspase-independent forms of cell death have also been described, often involving the release of mitochondrial proteins such as AIF and EndoG.^30, 31, 63^ Upon release, AIF and EndoG translocate to the nucleus where they induce DNA fragmentation and chromosome condensation.^32, 33, 63^ Though caspase-independent, CD40L-induced MPSC1 cell death does not appear to involve AIF and/or EndoG. Rather, our RIP1 inhibitor (necrostatin-1) and siRNA findings suggest that CD40L induces necroptosis, a form of controlled necrosis characterized by a dependency on RIP1, RIP3 and MLKL when caspases, especially caspase-8, are inhibited.^34, 35, 64, 65, 66^ Indeed, the induction of necroptosis-like cell death by CD40 activation is further supported by our RIP3 (GSK'872) and MLKL (necrosulfonamide) inhibitor results. RIP1-mediated necroptosis is becoming increasingly recognized as an important form of caspase-independent cell death,^34, 67^ however, pro-apoptotic roles for RIP1 have also been described in caspase-dependent, death receptor-mediated cell killing.^68, 69^ In EJ bladder cancer cells, RIP1 has been shown to mediate CD40L-induced caspase-8 activation and apoptosis, the latter being partially inhibited by necrostatin-1 and completely abolished by pan-caspase inhibitor.^70^ Moreover, the relationship of RIP1 to necroptosis can also vary depending on the cellular context, as recent studies have demonstrated that RIP1 may inhibit rather than promote necroptosis.^71^ This variation likely reflects the complex regulatory roles and interactions of RIP1 with other proteins involved in necrosome formation and necroptotic cell death. Indeed, such variation could explain the discrepancy between our RIP1 inhibitor and siRNA results such that RIP1 still acts as a crucial scaffold for protein–protein interactions when cells are treated with inhibitor (necrostatin-1), whereas this scaffold function would be disrupted when treating cells with siRNA.^35, 72^ Future research will be required to characterize, in detail, the precise molecular determinants of CD40L-induced cell death in LGSCs.

In summary, we have shown that CD40 is upregulated in a significant proportion of LGSCs (including LGSC-derived MPSC1 and VOA1312 cells) compared with SBOTs. CD40 activation induces RIP1-dependent, necroptosis-like cell death in MPSC1 but not SBOT3.1 cells. These findings provide insight into the function and therapeutic potential of the CD40 system in LGSCs.

## Materials and Methods

### Cell culture

The SBOT3.1,^73, 74^ MPSC1^75^ and VOA1312^27^ cell lines were kindly provided by Dr. Nelly Auersperg (Department of Obstetrics and Gynaecology, University of British Columbia, Canada), Dr Ie-Ming Shih (Department of Pathology, Johns Hopkins Medical Institutions, USA) and Dr David G. Huntsman (Department of Pathology and Laboratory Medicine, University of British Columbia, Canada), respectively. SBOT3.1 and VOA1312 cells were grown in a 1 : 1 (v/v) mixture of M199/MCDB105 medium (Sigma-Aldrich, Oakville, ON, Canada) supplemented with 10% fetal bovine serum (Hyclone Laboratories Inc., Logan, UT, USA). MPSC1 cells were maintained in RPMI 1640 medium (Invitrogen, Burlington, ON, Canada) supplemented with 10% fetal bovine serum. Cells were cultured at 37 °C in a humidified atmosphere containing 5% CO_2_ and 95% air.

### Frozen tissue samples

Frozen samples of primary tissue were obtained from the Ovarian Cancer Canada Tumor Bank with informed patient consent following approval from the University of British Columbia and British Columbia Cancer Agency Research Ethics Board. A cube of tissue was quickly removed from the cryovial, minced using a scalpel blade and transferred to a tube containing cell lysis buffer (Cell Signaling Technology, Danvers, MA, USA) with protease inhibitor cocktail (Sigma-Aldrich). Lysates were passed at least five times each through 18- and 22-gauge needles. Extracts were centrifuged at 20 000 × *g* for 10 min at 4 °C to remove cellular debris and supernatants were transferred to a clean microcentrifuge tube. Samples were stored at –80 °C until assayed by western blot as described below.

### Antibodies and reagents

Mouse monoclonal anti-*α*-Tubulin, goat polyclonal anti-actin (C-11) and rabbit polyclonal anti-CD40 (N-16) antibodies were obtained from Santa Cruz Biotechnology (Santa Cruz, CA, USA). Polyclonal anti-caspase-3 and anti-RIP1 antibodies were obtained from Cell Signaling Technology. Mouse monoclonal agonistic anti-CD40 (Clone # 82111) antibody was purchased from R&D Systems (Minneapolis, MN, USA). Horseradish peroxidase-conjugated goat anti-mouse IgG and goat anti-rabbit IgG were obtained from Bio-Rad Laboratories (Hercules, CA, USA). Recombinant human sCD40 ligand (CD40L) was obtained from Peprotech (Rocky Hill, NJ, USA). 3-(4,5-Dimethylthiazol-2-yl)-2,5-diphenyltetrazolium bromide (MTT), necrostatin-1 and 1-MT were purchased from Sigma-Aldrich. Boc-D-FMK was purchased from Abcam (Toronto, ON, Canada). GSK'872 and necrosulfonamide were purchased from Millipore (Etobicoke, ON, Canada).

### SiRNA transfection

To knockdown endogenous CD40, caspase-3, AIF, EndoG or RIP1, cells were transfected with 50 nM ON-TARGET*plus* SMARTpool siRNA or ON-TARGET*plus* Non-targeting Control Pool (Dharmacon, Lafayette, CO, USA) using Lipofectamine RNAiMAX (Invitrogen, Burlington, ON, USA).

### Western blot analysis

Cells were washed with cold PBS and lysed in lysis buffer (Cell Signaling Technology) containing protease inhibitor cocktail (Sigma-Aldrich). Extracts were centrifuged at 20 000 × *g* for 10 min at 4 °C and protein concentrations were determined using the DC Protein Assay (Bio-Rad Laboratories) with BSA as the standard. Equal amounts of protein were separated by SDS-polyacrylamide gel electrophoresis and transferred to polyvinylidene fluoride membranes. After blocking with Tris-buffered saline containing 5% non-fat dry milk for 1 h, the membranes were incubated overnight at 4 °C with primary antibodies followed by incubation with peroxidase-conjugated secondary antibody. Immunoreactive bands were detected using enhanced chemiluminescent substrate (Pierce, Rockford, IL, USA) followed by exposure to CL-XPosure film (Thermo Fisher, Waltham, MA, USA). Films were scanned and quantified by densitometry using Scion image software (Scion Corp., Frederick, MD, USA). CD40 and cleaved caspase-3 levels were normalized to *α*-tubulin. Alternatively, CD40 levels in primary tumor samples were normalized to actin.

### Immunohistochemistry

Formalin-fixed, paraffin-embedded tumor samples were assessed for CD40 expression. Sections were deparaffinized in xylene, rehydrated through graded alcohol, and processed for wet heat-induced antigen retrieval in a steamer for 20 min with a modified citrate buffer (pH 6.1; Dako, Burlington, ON, Canada). Endogenous peroxidase activity was quenched with 3% hydrogen peroxide in PBS for 30 min. Sections were blocked with serum-free protein block (Dako) for 30 min at room temperature, and then incubated overnight at 4 °C with polyclonal anti-CD40 (Abcam, Ab13545) diluted 1 : 250 in a serum-free protein block. Immunoreactivity was detected with the LSAB+HRP System (Dako) and 3,3′-diaminobenzidine chromogen solution (Dako). Slides were counterstained with hematoxylin (Sigma), dehydrated through graded alcohol to xylene, mounted with xylene-based mounting medium and evaluated by light microscopy.

### Reverse transcription quantitative real-time PCR (RT-qPCR)

Total RNA was extracted using TRIzol Reagent (Invitrogen) according to the manufacturer's instructions. Reverse transcription was performed with 3 *μ*g RNA, random primers and M-MLV reverse transcriptase (Promega, Madison, WI, USA). RT-qPCR was performed using an Applied Biosystems (Burlington, ON, Canada) 7300 Real-Time PCR System equipped with 96-well optical reaction plates. Each 20 *μ*l reaction contained 1 × SYBR Green PCR Master Mix (Applied Biosystems), 100 ng cDNA and 250 nM of each specific primer. The primers used for SYBR Green RT-qPCR were: CD40, 5′-CTG TTT GCC ATC CTC TTG GT-3′ (sense) and 5′-CGA CTC TCT TTG CCA TCC TC-3′ (antisense); CD40L, 5′-ATT GGG TCA GCA CTT TTT GC-3′ (sense) and 5′-TCA CAA AGC CTT CAA ACT GG-3′ (antisense); and GAPDH, 5′-GAG TCA ACG GAT TTG GTC GT-3′ (sense) and 5′-GAC AAG CTT CCC GTT CTC AG-3′ (antisense). The specificity of each assay was validated by dissociation curve analysis and agarose gel electrophoresis of PCR products. Assay performance was validated by evaluating amplification efficiencies by means of calibration curves, and ensuring that the plot of log input amount *versus* ΔCq has a slope <|0.1|. Alternatively, TaqMan gene expression assays were used for AIF, EndoG and GAPDH (Hs00377585_m1, Hs01035290_m1 and Hs02758991_g1, respectively; Applied Biosystems). Each 20 *μ*l TaqMan reaction contained 1 × TaqMan Gene Expression Master Mix (Applied Biosystems), 100 ng cDNA and 1 × TaqMan gene expression assay (containing primers and probe). The PCR parameters for SYBR Green and TaqMan RT-qPCR were 50 °C for 2 min, 95 °C for 10 min, and 40 cycles of 95 °C for 15 s and 60 °C for 1 min. All RT-qPCR results represent the mean of at least three separate experiments and each sample was assayed in triplicate. Relative quantification of mRNA levels was performed by the comparative Cq method with GAPDH as the reference gene and using the formula 2^–ΔΔCq^.

### MTT and Trypan blue exclusion assays

For the MTT assay, cells were seeded at a density of 2 × 10^4^ cells/well in 48-well plates and treated as described. MTT was added to a final concentration of 0.5 mg/ml, the cells were incubated for 4 h and the medium was removed. DMSO was added to each well and absorbances were measured at 490 nm using a microplate reader. For the Trypan blue exclusion assay, cells were seeded at a density of 5 × 10^4^ cells/well in 12-well plates and treated as described. Viable cell numbers were counted by Trypan blue dye exclusion using a hemocytometer. Results are expressed as a percentage relative to vehicle-treated control.

### Statistical analysis

Results are presented as the mean±S.E.M. of at least three separate experiments, and were analyzed by *t*-test or one-way ANOVA followed by Student-Newman-Keuls multiple comparison test using PRISM software (GraphPad Software, Inc., San Diego, CA, USA). Significant differences were defined as *P*<0.05.

## Abbreviations

AIF: apoptosis-inducing factor
CD40L: CD40 ligand
EndoG: endonuclease G
HGSCs: high-grade serous carcinomas
LGSCs: low-grade serous carcinomas
1-MT: 1-methyl-L-tryptophan
RIP1: receptor-interacting protein 1
RIP3: receptor-interacting protein 3
MLKL: mixed lineage kinase domain-like
RT-qPCR: reverse transcription quantitative real-time PCR
SBOTs: serous borderline ovarian tumors
siRNA: small interfering RNA

## Notes

The authors declare no conflict interest.

## References

[bib1] Crispens MA Bodurka D Deavers M Lu K Silva EG Gershenson DM Response and survival in patients with progressive or recurrent serous ovarian tumors of low malignant potential Obstet Gynecol 2002 99 3 10 11777502 10.1016/s0029-7844(01)01649-0

[bib2] Landen CN Jr Birrer MJ Sood AK Early events in the pathogenesis of epithelial ovarian cancer J Clin Oncol 2008 26 995 1005 18195328 10.1200/JCO.2006.07.9970

[bib3] Silva EG Gershenson DM Malpica A Deavers M The recurrence and the overall survival rates of ovarian serous borderline neoplasms with noninvasive implants is time dependent Am J Surg Pathol 2006 30 1367 1371 17063075 10.1097/01.pas.0000213294.81154.95

[bib4] Shih IeM Kurman RJ Ovarian tumorigenesis: a proposed model based on morphological and molecular genetic analysis Am J Pathol 2004 164 1511 1518 15111296 10.1016/s0002-9440(10)63708-x PMC1615664

[bib5] Singer G Kurman RJ Chang HW Cho SK Shih IeM Diverse tumorigenic pathways in ovarian serous carcinoma Am J Pathol 2002 160 1223 1228 11943707 10.1016/s0002-9440(10)62549-7 PMC1867233

[bib6] Cheng JC Auersperg N Leung PC EGF-induced EMT and invasiveness in serous borderline ovarian tumor cells: a possible step in the transition to low-grade serous carcinoma cells PLoS One 2012 7 e34071 22479527 10.1371/journal.pone.0034071 PMC3316602

[bib7] Cheng JC Auersperg N Leung PC TGF-beta induces serous borderline ovarian tumor cell invasion by activating EMT but triggers apoptosis in low-grade serous ovarian carcinoma cells PloS one 2012 7 e42436 22905131 10.1371/journal.pone.0042436 PMC3419689

[bib8] Cheng JC Auersperg N Leung PC Inhibition of p53 induces invasion of serous borderline ovarian tumor cells by accentuating PI3K/Akt-mediated suppression of E-cadherin Oncogene 2011 30 1020 1031 20972462 10.1038/onc.2010.486

[bib9] Cheng JC Auersperg N Leung PC Inhibition of p53 represses E-cadherin expression by increasing DNA methyltransferase-1 and promoter methylation in serous borderline ovarian tumor cells Oncogene 2011 30 3930 3942 21478913 10.1038/onc.2011.117

[bib10] Gershenson DM Sun CC Bodurka D Coleman RL Lu KH Sood AK Recurrent low-grade serous ovarian carcinoma is relatively chemoresistant Gynecol Oncol 2009 114 48 52 19361839 10.1016/j.ygyno.2009.03.001

[bib11] van Kooten C Banchereau J CD40-CD40 ligand J Leukoc Biol 2000 67 2 17 10647992 10.1002/jlb.67.1.2

[bib12] Grewal IS Flavell RA CD40 and CD154 in cell-mediated immunity Annu Rev Immunol 1998 16 111 135 9597126 10.1146/annurev.immunol.16.1.111

[bib13] Fonsatti E Maio M Altomonte M Hersey P Biology and clinical applications of CD40 in cancer treatment Semin Oncol 2010 37 517 523 21074067 10.1053/j.seminoncol.2010.09.002

[bib14] Gomes EM Rodrigues MS Phadke AP Butcher LD Starling C Chen S Antitumor activity of an oncolytic adenoviral-CD40 ligand (CD154) transgene construct in human breast cancer cells Clin Cancer Res 2009 15 1317 1325 19228733 10.1158/1078-0432.CCR-08-1360

[bib15] Wang X Chen B Xu W Liu S Zhao W Wu J Combined effects of klotho and soluble CD40 ligand on A549 lung cancer cells Oncol Rep 2011 25 1465 1472 21308354 10.3892/or.2011.1178

[bib16] Fiumara P Younes A CD40 ligand (CD154) and tumour necrosis factor-related apoptosis inducing ligand (Apo-2L) in haematological malignancies Br J Haematol 2001 113 265 274 11380390 10.1046/j.1365-2141.2001.02593.x

[bib17] Planken EV Dijkstra NH Willemze R Kluin-Nelemans JC Proliferation of B cell malignancies in all stages of differentiation upon stimulation in the 'CD40 system' Leukemia 1996 10 488 493 8642867

[bib18] Baxendale AJ Dawson CW Stewart SE Mudaliar V Reynolds G Gordon J Constitutive activation of the CD40 pathway promotes cell transformation and neoplastic growth Oncogene 2005 24 7913 7923 16091748 10.1038/sj.onc.1208929

[bib19] Homig-Holzel C Hojer C Rastelli J Casola S Strobl LJ Muller W Constitutive CD40 signaling in B cells selectively activates the noncanonical NF-kappaB pathway and promotes lymphomagenesis J Exp Med 2008 205 1317 1329 18490492 10.1084/jem.20080238 PMC2413030

[bib20] Zhou Y He J Gou LT Mu B Liao WC Ma C Expression of CD40 and growth-inhibitory activity of CD40 agonist in ovarian carcinoma cells Cancer Immunol Immunother 2012 61 1735 1743 22406982 10.1007/s00262-011-1194-0 PMC11029153

[bib21] Gallagher NJ Eliopoulos AG Agathangelo A Oates J Crocker J Young LS CD40 activation in epithelial ovarian carcinoma cells modulates growth, apoptosis, and cytokine secretion Mol Pathol 2002 55 110 120 11950960 10.1136/mp.55.2.110 PMC1187159

[bib22] Hakkarainen T Hemminki A Pereboev AV Barker SD Asiedu CK Strong TV CD40 is expressed on ovarian cancer cells and can be utilized for targeting adenoviruses Clin Cancer Res 2003 9 619 624 12576427

[bib23] Toutirais O Gervais A Cabillic F Le Gallo M Coudrais A Leveque J Effects of CD40 binding on ovarian carcinoma cell growth and cytokine production *in vitro* Clin Exp Immunol 2007 149 372 377 17565609 10.1111/j.1365-2249.2007.03426.x PMC1941941

[bib24] Melichar B Patenia R Gallardo S Melicharova K Hu W Freedman RS Expression of CD40 and growth-inhibitory activity of CD40 ligand in ovarian cancer cell lines Gynecol Oncol 2007 104 707 713 17166566 10.1016/j.ygyno.2006.10.056

[bib25] Ghamande S Hylander BL Oflazoglu E Lele S Fanslow W Repasky EA Recombinant CD40 ligand therapy has significant antitumor effects on CD40-positive ovarian tumor xenografts grown in SCID mice and demonstrates an augmented effect with cisplatin Cancer Res 2001 61 7556 7562 11606394

[bib26] Shih IeM Chen L Wang CC Gu J Davidson B Cope L Distinct DNA methylation profiles in ovarian serous neoplasms and their implications in ovarian carcinogenesis Am J Obstet Gynecol 2010 203 584 e581 522 20965493 10.1016/j.ajog.2010.08.003 PMC2993872

[bib27] Anglesio MS Wiegand KC Melnyk N Chow C Salamanca C Prentice LM Type-specific cell line models for type-specific ovarian cancer research PLoS One 2013 8 e72162 24023729 10.1371/journal.pone.0072162 PMC3762837

[bib28] Luthi AU Martin SJ The CASBAH: a searchable database of caspase substrates Cell Death Differ 2007 14 641 650 17273173 10.1038/sj.cdd.4402103

[bib29] Timmer JC Salvesen GS Caspase substrates Cell Death Differ 2007 14 66 72 17082814 10.1038/sj.cdd.4402059

[bib30] Mayer B Oberbauer R Mitochondrial regulation of apoptosis News in Physiological Sciences 2003 18 89 94 12750442 10.1152/nips.01433.2002

[bib31] Zamzami N Kroemer G The mitochondrion in apoptosis: how Pandora's box opens Nat Rev Mol Cell Biol 2001 2 67 71 11413468 10.1038/35048073

[bib32] Susin SA Lorenzo HK Zamzami N Marzo I Snow BE Brothers GM Molecular characterization of mitochondrial apoptosis-inducing factor Nature 1999 397 441 446 9989411 10.1038/17135

[bib33] Li LY Luo X Wang X Endonuclease G is an apoptotic DNase when released from mitochondria Nature 2001 412 95 99 11452314 10.1038/35083620

[bib34] Vandenabeele P Galluzzi L Vanden Berghe T Kroemer G Molecular mechanisms of necroptosis: an ordered cellular explosion Nat Rev Mol Cell Biol 2010 11 700 714 20823910 10.1038/nrm2970

[bib35] de Almagro MC Vucic D Necroptosis: Pathway diversity and characteristics Semin Cell Dev Biol 2015 39 56 62 25683283 10.1016/j.semcdb.2015.02.002

[bib36] Degterev A Maki JL Yuan J Activity and specificity of necrostatin-1, small-molecule inhibitor of RIP1 kinase Cell Death Differ 2013 20 366 23197295 10.1038/cdd.2012.133 PMC3554332

[bib37] Takahashi N Duprez L Grootjans S Cauwels A Nerinckx W DuHadaway JB Necrostatin-1 analogues: critical issues on the specificity, activity and *in vivo* use in experimental disease models Cell Death Dis 2012 3 e437 23190609 10.1038/cddis.2012.176 PMC3542611

[bib38] Sun L Wang H Wang Z He S Chen S Liao D Mixed lineage kinase domain-like protein mediates necrosis signaling downstream of RIP3 kinase Cell 2012 148 213 227 22265413 10.1016/j.cell.2011.11.031

[bib39] Eliopoulos AG Davies C Knox PG Gallagher NJ Afford SC Adams DH CD40 induces apoptosis in carcinoma cells through activation of cytotoxic ligands of the tumor necrosis factor superfamily Mol Cell Biol 2000 20 5503 5515 10891490 10.1128/mcb.20.15.5503-5515.2000 PMC86001

[bib40] Tong AW Papayoti MH Netto G Armstrong DT Ordonez G Lawson JM Growth-inhibitory effects of CD40 ligand (CD154) and its endogenous expression in human breast cancer Clin Cancer Res 2001 7 691 703 11297266

[bib41] Hess S Engelmann H A novel function of CD40: induction of cell death in transformed cells J Exp Med 1996 183 159 167 8551219 10.1084/jem.183.1.159 PMC2192400

[bib42] von Leoprechting A van der Bruggen P Pahl HL Aruffo A Simon JC Stimulation of CD40 on immunogenic human malignant melanomas augments their cytotoxic T lymphocyte-mediated lysis and induces apoptosis Cancer Res 1999 59 1287 1294 10096561

[bib43] Hirano A Longo DL Taub DD Ferris DK Young LS Eliopoulos AG Inhibition of human breast carcinoma growth by a soluble recombinant human CD40 ligand Blood 1999 93 2999 3007 10216096

[bib44] Hassan SB Sorensen JF Olsen BN Pedersen AE Anti-CD40-mediated cancer immunotherapy: an update of recent and ongoing clinical trials Immunopharmacol Immunotoxicol 2014 36 96 104 24555495 10.3109/08923973.2014.890626

[bib45] Lanzavecchia A Immunology. Licence to kill Nature 1998 393 413 414 9623994 10.1038/30845

[bib46] Todryk SM Tutt AL Green MH Smallwood JA Halanek N Dalgleish AG CD40 ligation for immunotherapy of solid tumours J Immunol Methods 2001 248 139 147 11223075 10.1016/s0022-1759(00)00349-5

[bib47] Vonderheide RH Bajor DL Winograd R Evans RA Bayne LJ Beatty GL CD40 immunotherapy for pancreatic cancer Cancer Immunol Immunother 2013 62 949 954 23589109 10.1007/s00262-013-1427-5 PMC3731141

[bib48] Lum HD Buhtoiarov IN Schmidt BE Berke G Paulnock DM Sondel PM *In vivo* CD40 ligation can induce T-cell-independent antitumor effects that involve macrophages J Leukoc Biol 2006 79 1181 1192 16565324 10.1189/jlb.0405191

[bib49] Beatty GL Chiorean EG Fishman MP Saboury B Teitelbaum UR Sun W CD40 agonists alter tumor stroma and show efficacy against pancreatic carcinoma in mice and humans Science 2011 331 1612 1616 21436454 10.1126/science.1198443 PMC3406187

[bib50] Coughlin CM Vance BA Grupp SA Vonderheide RH RNA-transfected CD40-activated B cells induce functional T-cell responses against viral and tumor antigen targets: implications for pediatric immunotherapy Blood 2004 103 2046 2054 14630810 10.1182/blood-2003-07-2379

[bib51] Turner JG Rakhmilevich AL Burdelya L Neal Z Imboden M Sondel PM Anti-CD40 antibody induces antitumor and antimetastatic effects: the role of NK cells J Immunol 2001 166 89 94 11123280 10.4049/jimmunol.166.1.89

[bib52] Rakhmilevich AL Alderson KL Sondel PM T-cell-independent antitumor effects of CD40 ligation Int Rev Immunol 2012 31 267 278 22804571 10.3109/08830185.2012.698337 PMC3537496

[bib53] Galluzzi L Vitale I Abrams JM Alnemri ES Baehrecke EH Blagosklonny MV Molecular definitions of cell death subroutines: recommendations of the Nomenclature Committee on Cell Death 2012 Cell Death Differ 2012 19 107 120 21760595 10.1038/cdd.2011.96 PMC3252826

[bib54] Kroemer G Galluzzi L Vandenabeele P Abrams J Alnemri ES Baehrecke EH Classification of cell death: recommendations of the Nomenclature Committee on Cell Death 2009 Cell Death Differ 2009 16 3 11 18846107 10.1038/cdd.2008.150 PMC2744427

[bib55] Elmore S Apoptosis: a review of programmed cell death Toxicol Pathol 2007 35 495 516 17562483 10.1080/01926230701320337 PMC2117903

[bib56] Yi CH Yuan J The Jekyll and Hyde functions of caspases Dev Cell 2009 16 21 34 19154716 10.1016/j.devcel.2008.12.012 PMC2850564

[bib57] Connolly PF Jager R Fearnhead HO New roles for old enzymes: killer caspases as the engine of cell behavior changes Front Physiol 2014 5 149 24795644 10.3389/fphys.2014.00149 PMC3997007

[bib58] Zermati Y Garrido C Amsellem S Fishelson S Bouscary D Valensi F Caspase activation is required for terminal erythroid differentiation J Exp Med 2001 193 247 254 11208865 10.1084/jem.193.2.247 PMC2193347

[bib59] Carlile GW Smith DH Wiedmann M Caspase-3 has a nonapoptotic function in erythroid maturation Blood 2004 103 4310 4316 14976035 10.1182/blood-2003-09-3362

[bib60] Woo M Hakem R Furlonger C Hakem A Duncan GS Sasaki T Caspase-3 regulates cell cycle in B cells: a consequence of substrate specificity Nat Immunol 2003 4 1016 1022 12970760 10.1038/ni976

[bib61] Janzen V Fleming HE Riedt T Karlsson G Riese MJ Lo Celso C Hematopoietic stem cell responsiveness to exogenous signals is limited by caspase-3 Cell Stem Cell 2008 2 584 594 18522851 10.1016/j.stem.2008.03.012 PMC2991117

[bib62] Fujita J Crane AM Souza MK Dejosez M Kyba M Flavell RA Caspase activity mediates the differentiation of embryonic stem cells Cell Stem Cell 2008 2 595 601 18522852 10.1016/j.stem.2008.04.001 PMC2494585

[bib63] Tait SW Green DR Caspase-independent cell death: leaving the set without the final cut Oncogene 2008 27 6452 6461 10.1038/onc.2008.311 PMC2635930 18955972

[bib64] Cho YS Challa S Moquin D Genga R Ray TD Guildford M Phosphorylation-driven assembly of the RIP1-RIP3 complex regulates programmed necrosis and virus-induced inflammation Cell 2009 137 1112 1123 19524513 10.1016/j.cell.2009.05.037 PMC2727676

[bib65] He S Wang L Miao L Wang T Du F Zhao L Receptor interacting protein kinase-3 determines cellular necrotic response to TNF-alpha Cell 2009 137 1100 1111 19524512 10.1016/j.cell.2009.05.021

[bib66] Zhang DW Shao J Lin J Zhang N Lu BJ Lin SC RIP3, an energy metabolism regulator that switches TNF-induced cell death from apoptosis to necrosis Science 2009 325 332 336 19498109 10.1126/science.1172308

[bib67] Festjens N Vanden Berghe T Cornelis S Vandenabeele P RIP1, a kinase on the crossroads of a cell's decision to live or die Cell Death Differ 2007 14 400 410 17301840 10.1038/sj.cdd.4402085

[bib68] Abhari BA Cristofanon S Kappler R von Schweinitz D Humphreys R Fulda S RIP1 is required for IAP inhibitor-mediated sensitization for TRAIL-induced apoptosis via a RIP1/FADD/caspase-8 cell death complex Oncogene 2013 32 3263 3273 22890322 10.1038/onc.2012.337

[bib69] Lin Y Devin A Rodriguez Y Liu ZG Cleavage of the death domain kinase RIP by caspase-8 prompts TNF-induced apoptosis Genes Dev 1999 13 2514 2526 10521396 10.1101/gad.13.19.2514 PMC317073

[bib70] Knox PG Davies CC Ioannou M Eliopoulos AG The death domain kinase RIP1 links the immunoregulatory CD40 receptor to apoptotic signaling in carcinomas J Cell Biol 2011 192 391 399 21282461 10.1083/jcb.201003087 PMC3101101

[bib71] Kearney CJ Cullen SP Clancy D Martin SJ RIPK1 can function as an inhibitor rather than an initiator of RIPK3-dependent necroptosis FEBS J 2014 281 4921 4934 25195660 10.1111/febs.13034

[bib72] Weiss WA Taylor SS Shokat KM Recognizing and exploiting differences between RNAi and small-molecule inhibitors Nat Chem Biol 2007 3 739 744 10.1038/nchembio1207-739 PMC2924165 18007642

[bib73] Woo MM Salamanca CM Symowicz J Stack MS Miller DM Leung PC SV40 early genes induce neoplastic properties in serous borderline ovarian tumor cells Gynecol Oncol 2008 111 125 131 18678400 10.1016/j.ygyno.2008.06.021

[bib74] Woo MM Salamanca CM Miller M Symowicz J Leung PC Oliveira C Serous borderline ovarian tumors in long-term culture: phenotypic and genotypic distinction from invasive ovarian carcinomas Int J Gynecol Cancer 2008 18 1234 1247 18217967 10.1111/j.1525-1438.2007.01171.x

[bib75] Pohl G Ho CL Kurman RJ Bristow R Wang TL Shih IeM Inactivation of the mitogen-activated protein kinase pathway as a potential target-based therapy in ovarian serous tumors with KRAS or BRAF mutations Cancer Res 2005 65 1994 2000 15753399 10.1158/0008-5472.CAN-04-3625

