# Supplementary figures and images for: CD40 ligand induces RIP1-dependent, necroptosis-like cell death in low-grade serous but not serous borderline ovarian tumor cells
Source: Cell Death Dis. 2015 Aug 27;6(8):e1864–. doi: 10.1038/cddis.2015.229 (PMC4558516; doi:10.1038/cddis.2015.229)

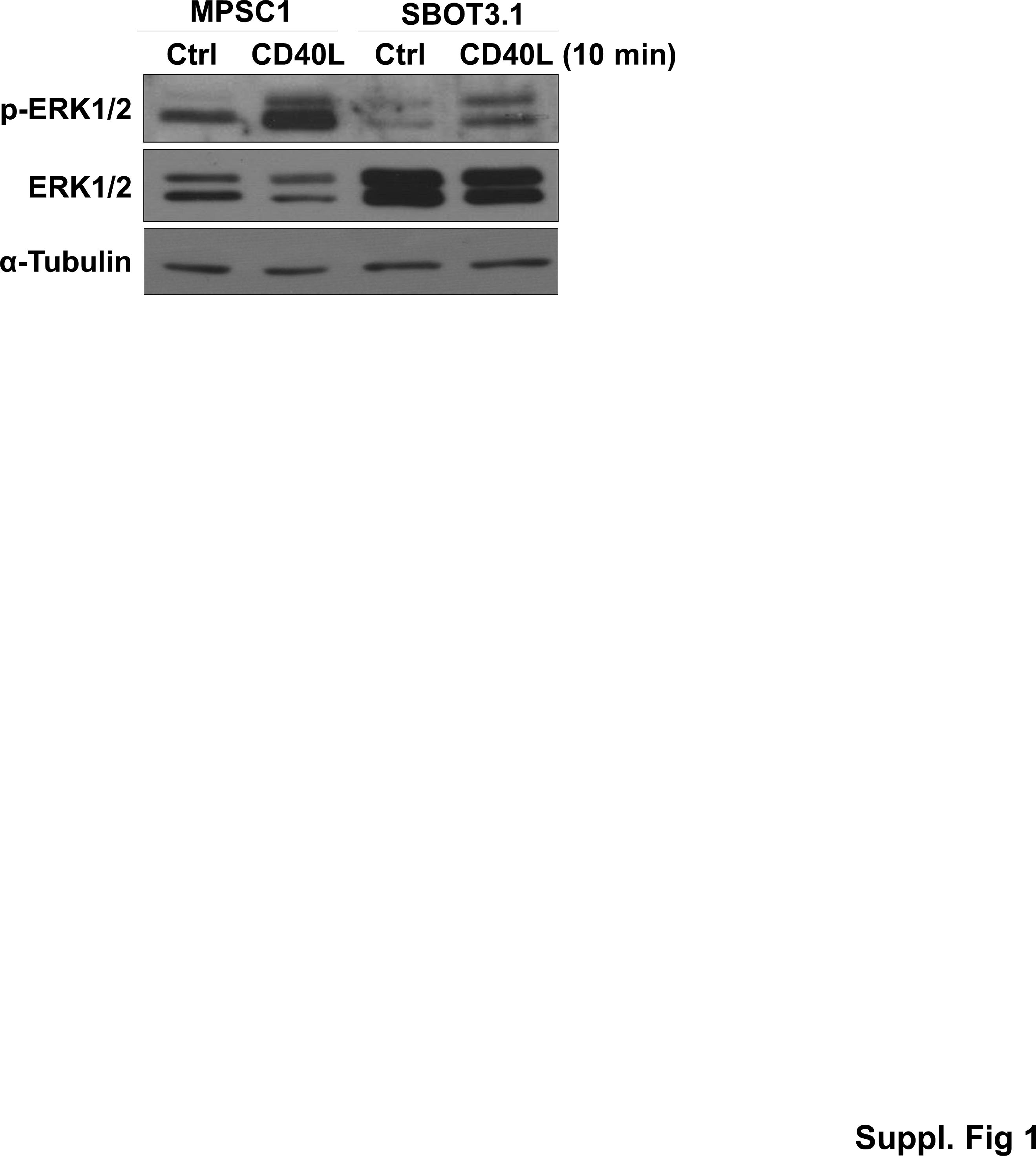

Supplement: Supplementary Figure 1 [file cddis2015229x1.tif]
