# Supplementary material for: CD40 ligand induces RIP1-dependent, necroptosis-like cell death in low-grade serous but not serous borderline ovarian tumor cells
Source: Cell Death Dis. 2015 Aug 27;6(8):e1864–. doi: 10.1038/cddis.2015.229 (PMC4558516; doi:10.1038/cddis.2015.229)
Supplement: Supplementary Figure Legend [file cddis2015229x2.docx]

**Supplementary Figure Legend**

**S Figure 1.** CD40L activates ERK1/2 signaling pathway in LGSC-derived MPSC1 cells and SBOT-derived SBOT3.1 cells. MPSC1 and SBOT3.1 cells were treated with 500 ng/mL CD40L for 10 min and the phosphorylation levels of ERK1/2 were determined by western blot using antibody specific for phosphorylated forms of ERK1/2 (p-ERK1/2). Membranes were stripped and reprobed with antibodies to total ERK1/2 and α-Tubulin.
